# Zinc Sorption on Modified Waste Poly(methyl methacrylate)

**DOI:** 10.3390/ma10070755

**Published:** 2017-07-06

**Authors:** Agata Jakóbik-Kolon, Andrzej Milewski, Dominik Zdybał, Krzysztof Mitko, Ewa Laskowska, Anna Mielańczyk, Joanna Bok-Badura

**Affiliations:** Faculty of Chemistry, Silesian University of Technology, Krzywoustego 6, 44-100 Gliwice, Poland; andrzej.milewski@polsl.pl (A.Mil.); zdybal_dominik@wp.pl (D.Z.); krzysztof.mitko@polsl.pl (K.M.); ewa.laskowska@polsl.pl (E.L.); anna.mielanczyk@polsl.pl (A.Mie.); joanna.bok-badura@polsl.pl (J.B.-B)

**Keywords:** poly(methyl methacrylate), waste, zinc sorption, poly(methacrylic salts)

## Abstract

The new one-pot hydrolysis-crosslinking reaction was used to synthesize a new, waste poly(methyl methacrylate) (PMMA)-based material for zinc(II) ions removal. The alkaline hydrolysis of PMMA in diethylene glycol diethyl ether was used to obtain polymer matrix and it was then crosslinked with Ca and Mg ions to obtain the sorbent. As a result, the macroporous materials were obtained with a yield of 87% when waste PMMA was used, and about 95% when the commercial PMMAs were used. The degree of hydrolysis was similar, from 32% to 35%. New materials were then tested for their affinity towards zinc(II) ions. Two kinetic models (pseudo-first and pseudo-second order), as well as two isotherms (Langmuir and Freundlich), were used to describe the kinetics and equilibrium of zinc(II) ion sorption on the studied materials, respectively. All the prepared PMMA-based sorbents showed similar or higher sorption capacity (q up to 87.7 mg/g) compared to commercially available materials in a broad pH range (4–7). The study shows sorption was fast—above 80% of equilibrium capacity was achieved after ca. 0.5 h. Presented results show that waste PMMA may be an interesting raw material for the preparation of sorbents for zinc(II) ions removal.

## 1. Introduction

Today, the poly(methyl methacrylate) (PMMA) is commonly used in many applications, which include ordinary materials and special purpose plastics. PMMA is a transparent thermoplastic polymer, widely used as a substitute for inorganic glass, because it is lightweight, shows high impact strength and shatter-resistance, and exhibits favorable processing conditions. Moreover, this polymer has very good weather and scratch resistance. Thus, PMMA plates are used instead of glass as construction and building materials e.g., in parts of glass-cases, bus-stops, windows and doors, as well as some elements of cars in the automotive industry. Moreover, PMMA is a promising polymer for applications in optical, sensor, analytical and conductive devices. PMMA and its copolymers can also be used as a matrix in the drug delivery by electro-diffusion or electro-osmotic flow, as the battery electrolytes, or as the heavy metals sorbents.

PMMA is one of the few polymers [1,2,3] which can be recycled with good yield to monomers by pyrolysis; however, thermal decomposition to monomer is not only highly energy consuming, but also requires additional purification and stabilization of the obtained methyl methacrylate [4]. In our alternative strategy, waste PMMA is treated chemically and reused. In this case, a reasonably effective method is a hydrolysis reaction, which can be catalyzed using a strong acid (H_2_SO_4_) [1,5] or base (potassium hydroxide, sodium hydroxide, or its alcoholates) [6,7]; the PMMA copolymers, such as poly(methyl methacrylate)-*co*-poly(methacrylic acid) or poly(methyl methacrylate)-*co*-poly(methacrylic salts), are obtained, respectively.

The spatial arrangement of monomeric units is a crucial factor that determines the hydrolysis rate of PMMA. In fact, the tacticity is far more important than the molecular mass of a polymer chain. The characteristic fast hydrolysis of isotactic PMMA has been known for a long time [8]; however, there is a lack of consensus on the reason for this phenomena [9]. Unfortunately, industrial radical polymerisation of MMA results in atactic structure of high molecular mass, usually over 100,000 Da. Steric hindrance and local negative charge of carboxylate groups suppress the reaction rate [8]. In general, the acidic hydrolysis provides low hydrolysis reaction rate and is a time consuming path [5]. Despite the almost 70 years of polymer technology development, there is still a lack of the rapid and green method for basic poly(methyl methacrylate) hydrolysis or saponification. What is more, there is no published evidence for the one-pot hydrolysis-crosslinking procedure of conventional PMMA. Various reagents for alkaline hydrolysis or saponification were proposed, including ethylene glycol-potassium hydroxide [6], dioxane-methanol-potassium hydroxide [10], dioxane-PEG-potassium hydroxide [11], isopropanol-water-sodium hydroxide [12], DMSO-potassium hydroxide [13].

There is a strong pursuit toward novel, green reaction solvents for polymer synthesis and modification [14]. One class of the promising solvents is the glymes, ethers of ethylene glycols oligomers, which are immensely useful in laboratory scale synthesis, industrial processes and in ready-to-use products [15]. As some of them were found toxic, selection of appropriate glyme, including its impact on humans and the environment, is essential. Diethylene glycol diethyl ether (DEGDE) is a high boiling solvent (189 °C), which exhibits excellent solvency properties toward PMMA, polystyrene, poly(vinyl acetate), poly(ethylene glycols), is stable in basic conditions, and does not form unstable peroxide. Therefore, DEGDE was selected as a solvent medium for the modification reaction of poly(methyl methacrylate).

On the other hand, the removal of zinc, which is used in many branches of the industry as a component of batteries, paints, cosmetics, coatings, is an important issue. Because zinc may be accumulated in the living tissues, its excess may cause various diseases. A lot of methods, including precipitation, sedimentation, membrane processes, coagulation, flotation, electrochemical processes, adsorption, and ion exchange, have been proposed for heavy metal ions removal. Among them, adsorption is the most effective and economic, particularly if the concentration of the pollutant in wastewater is low. Various synthetic and commercially available resins have been proposed for zinc ions removal e.g., sulfonated resins such as Purolite C-100 MH [16], Amberlite IR-120 [17] and Dowex 50W [18] or Amberlite IRC-784 with iminodiacetic acid grups [19].

In our work, a new material for zinc(II) ions removal, based on the waste PMMA, have been synthesized and used. The new one-pot reaction of waste PMMA hydrolysis-crosslinking was proposed. Hydrolyzed material was next crosslinked with Ca and Mg ions to obtain the sorbent for zinc(II) ions removal. Such a solution has ecological and economical advantages: it helps in waste PMMA management and waste water purification. Interconnectivity of cells alter convective flow in materials structure occurs and it was found to be beneficial for rapid sorption of cooper ions on poly(methacrylic acid) hyderogel [20]. Macroporous materials have been obtained in this report, which resulted in fast equilibration of zinc sorption.

## 2. Results and Discussion

The waste polymer was confirmed to be PMMA by the ATR-IR method. The ceramic additives (ca. 0.6% w/w) were found to be TiO_2_. It was probably used as a pigment or opacifier since the used waste PMMA was not transparent. The waste polymeric material was checked on content of the non-ceramic additives in dried PMMA by ^1^H and ^13^C NMR.

**Figure 1:**
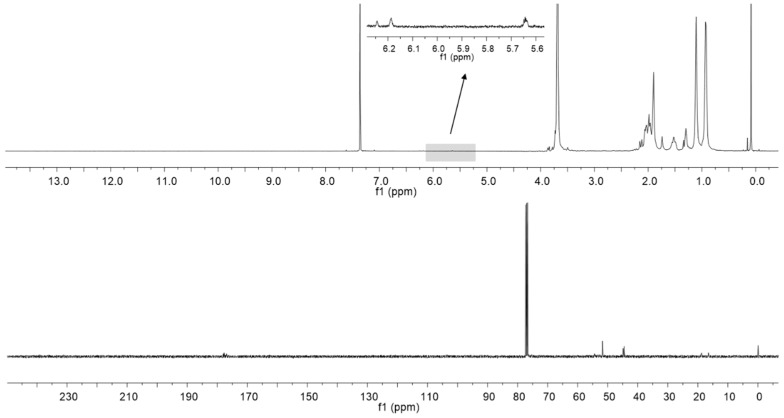
^1^H and ^13^C NMR spectra of waste poly(methyl methacrylate) (PMMA) with magnified monomer signals region.

In this case, only the monomer (as non-ceramic additive) content was confirmed by the two low signals between 5.6 and 6.2 ppm at the ^1^H NMR spectrum (Figure 1). On the ^1^H NMR spectrum, the signals from other additives have not been found, e.g., poly(vinyl alcohol), which would have been observed as shifts between 4.0 and 4.5 ppm. The results were also confirmed by ^13^C NMR spectrum (Figure 1). Molecular weight and dispersity indices were determined by size-exclusion chromatograph (SEC) to be 117,000 Da and 2.07, respectively.

### 2.1. Polymer Matrix Preparation from PMMA by the One Flask Hydrolysis-Crosslinking Reaction

In the preliminary studies, the PMMA acidic hydrolysis was tested with sulfuric acid using various solvents, in which the polymer was insoluble (carbon tetrachloride and hydrochloric acid) or soluble (toluene, dietyhylene glycol diethyl ether and dichloromethane). In these experiments, reaction mixture was heated under reflux at the boiling point of the solvent, with a catalytic agent. Experiments were stopped after 1 h, solvent was evaporated and product was dried to constant mass. Then, the degree of hydrolysis was determined by means of colorimetric titration. Although the processes were generally not effective, the homogenous mixtures provided best results. If the PMMA was not soluble in the reaction mixture, the material could not be moistened as well as when it was completely dissolved, therefore, the reaction with the polymer chain occurred only on the surface of a small compact particle. Sorbents prepared from such materials had poor sorption capacity towards zinc ions. Therefore, in the other set of preliminary experiments, the alkaline hydrolysis of PMMA was examined by using potassium hydroxide in the homogenous mixture only. The best results were obtained with dietyhylene glycol diethyl ether (DEGDE), which is a known stable solvent even at high pH values, making it an excellent solvent for reactions with strong bases or reactions that require high temperatures. Additionally, we have observed an increase in hydrolysis degree with the addition of dimethyl sulfoxide (DMSO) and diethylene glycol methyl ether (DEGME) into the polymer solution. DMSO allows for a higher degree of hydrolysis and was also found indispensable for allowing crosslinking to occur. DEGME provided higher potassium hydroxide solubility and better homogeneity of reaction mixture. Finally, the new, one-pot alkaline hydrolysis of PMMA was used to obtain polymer matrix (PM1–3, Table 1) and sorbent (PMMA1–6, Table 1). The synthesis and product characterization are shown in Table 1.

In these experiments, the waste PMMA was used to obtain the hydrolyzed products. Additionally, commercially available PMMA, although of higher molecular weight, was used as the reference. The lowest product yield was 87% (w/w) for a sample from the waste PMMA, which might have been caused by the broad distribution of Mw and the presence of low-mass fractions, soluble during material purification. For the commercial PMMAs, the product yields were similar (Table 1) and achieved ca. 95% (w/w), showing the molar weight of polymers had no important influence on this reaction. This hypothesis may be confirmed by the similar values of degree of hydrolysis, which were obtained and determined to be between 32 to 35% (w/w) of hydrolyzed groups.

Sorbent yields of PMMA1–6 were similar to yield of products PM1–3, because these procedures are based on a simple reaction between ions, and the total samples mass were saved.

**Figure 2:**
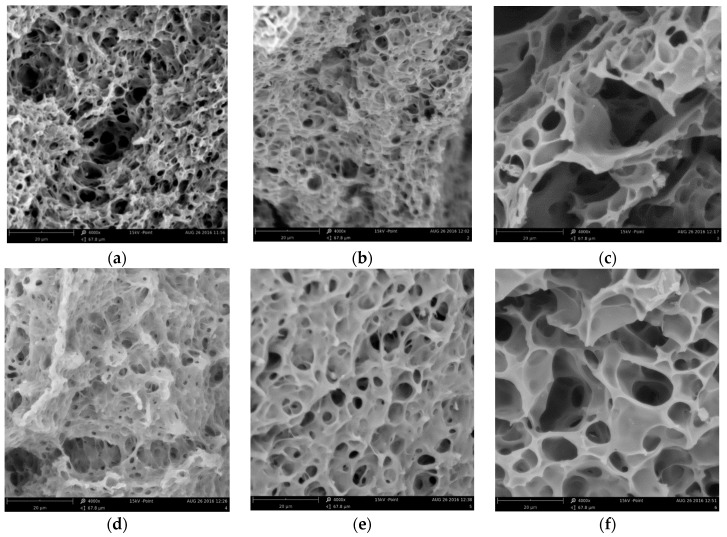
The SEM micrographs of PMMA-based sorbents (magnification 4000×): (**a**) PMMA1; (**b**) PMMA2; (**c**) PMMA3; (**d**) PMMA4; (**e**) PMMA5; (**f**) PMMA6.

The SEM micrographs (Figure 2) show that Mw of PMMA influence the pore size of the obtained sorbent—the size of the pores increase as the Mw decreases, which may be attributed to washing-off of the uncrosslinked fraction acting as an additional porogen. However, macroporous structure has been conserved in all specimens, which may strongly promote the kinetics of zinc removal. 

### 2.2. Zinc(II) Ions Sorption Studies—pH Dependence

**Figure 3:**
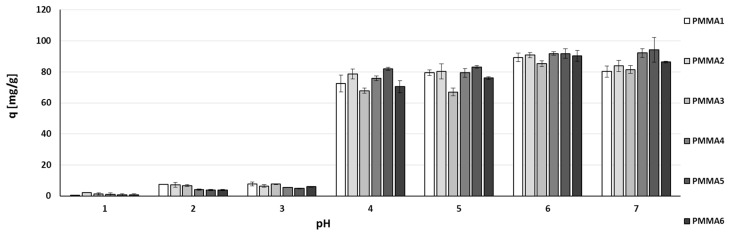
Comparison of sorption capacity of zinc ions at various pH values (1–7) on PMMA—based sorbents. Mass of sorbent 0.01 g, initial concentration of metal: *c* = 30 mg/L, volume of solution: 0.05 L, pH = 6, temperature 22 ± 1 °C, time of contact 24 h.

**Figure 4:**
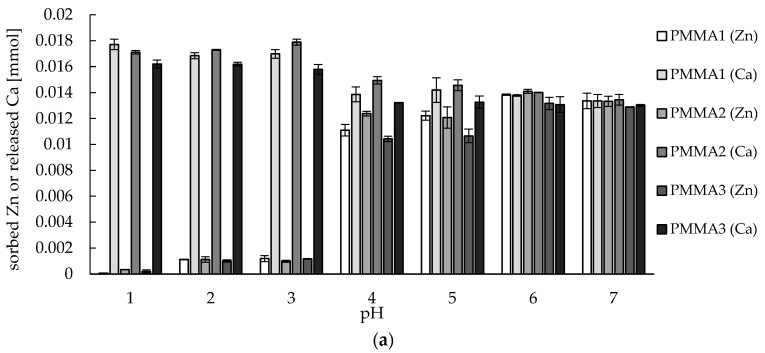

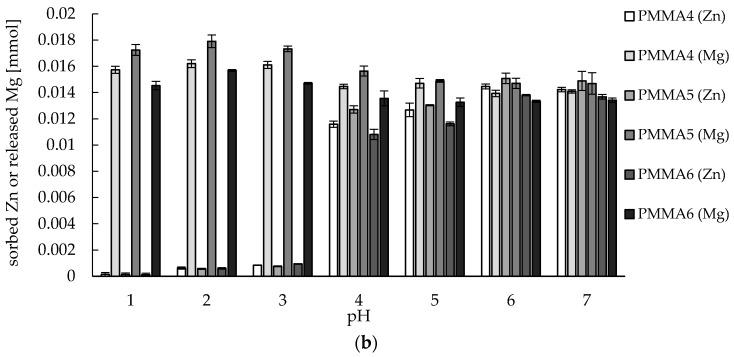
Comparison of amount of sorbed zinc(II) and amount of released (**a**) calcium(II), (**b**) magnesium(II) ions during sorption studies at various pH conditions. Mass of sorbent 0.01 g, initial concentration of metal: *c* = 30 mg/L, volume of solution: 0.05 L, pH = 6, temperature 22 ± 1 °C.

All PMMA-based sorbents showed very good sorption capacity (q) in a broad pH range (4–7) (Figure 3). Below pH 4, sorption capacities of studied materials drop significantly due to the competition of hydrogen ions with zinc(II) ions. Additionally, at lower pH, hydrogen ions are in excess and substitute the calcium and magnesium ions (Figure 4). As a result, carboxyl groups are created, being poorly dissociated in such conditions and thus mostly inert for metal ions. Sorbents prepared from waste material (PMMA3 and 6) have slightly lower, but still very high sorption capacity in comparison to sorbents prepared from pure polymers (PMMA1, 2, 4, 5). Generally, it can be stated that all sorbents, regardless the counter-cation (Ca, Mg), PMMA origin or molecular weight, have roughly the same sorption capacity, especially at pH = 6.

During the studies, apart from the zinc(II) ions sorption, the amount of calcium(II) and magnesium(II) ions released into the solution (in mmole) was measured. Next, the results were compared with the amount of zinc(II) ions (in mmole) sorbed on the PMMA-based materials (Figure 4). Equal number of mmoles of released Ca(II) or Mg(II) ions and mmoles of sorbed Zn(II) ions at higher pH values (6–7) proves the pure ion-exchange mechanism (Ca-Zn or Mg-Zn) of zinc sorption on studied materials. As mentioned above, at lower pH calcium(II) and magnesium(II), ions were replaced by hydronium ions and released into the solution. This replacement occurred partially at pH 4–5 and was significant at pH below 4, where amount of released Ca and Mg was even a few times greater than amounts of sorbed Zn.

### 2.3. Zinc(II) Ions Sorption Studies—Kinetics

**Figure 5:**
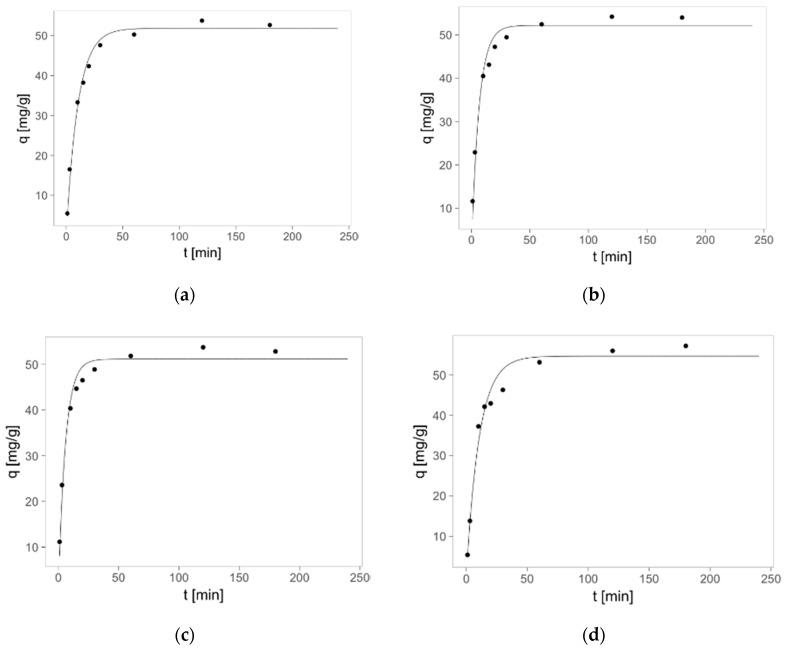

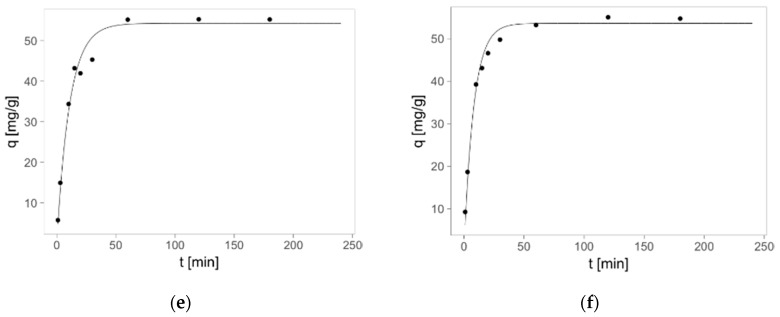
Adsorption kinetics of zinc(II) ions on PMMA—based sorbents: (**a**) PMMA1; (**b**) PMMA2; (**c**) PMMA3; (**d**) PMMA4; (**e**) PMMA5; (**f**) PMMA6 (pseudo-second order model). Mass of sorbent 0.01 g, initial concentration of metal: *c* = 30 mg/L, volume of solution: 0.02 L, pH = 6, temperature 22 ± 1 °C.

The sorption kinetics of Zn(II) ions on PMMA-based sorbents was analyzed using the two elementary kinetic models: pseudo-first order and pseudo-second order. The kinetic curves and parameters of these models are presented in Figure 5 and Table 2, respectively. The studies show that sorption in the investigated system is fast—above 80% of equilibrium capacity was achieved after ca. 0.5 h and 100% after ca. 1 h (Figure 5).

The pseudo-second order model fits better to the kinetic data (*R*^2^ > 0.99, Table 2), which corresponds to the chemisorption mechanism. It is in agreement with the proved above ion-exchange mechanism. As previously mentioned, the size of pores increases with PMMA molecular weight decrease. This also influences the kinetics of Zn(II) sorption—the rate constant increases with Mw decrease. The kind of ion (Mg, Ca) used for cross-linking also influences sorption kinetics of zinc(II) ions on prepared ion-exchangers. Thus, the highest rate constant was obtained for materials prepared from waste PMMA crosslinked with calcium(II) ions. The model allowed also for calculation of sorption capacity in equilibrium (*q*_m_ [mg/g])—the maximum amount zinc(II) ions [mg] adsorbed on one gram of sorbent at equilibrium in given conditions (sorbent dose, initial metal ions concentration, temperature). The values for various sorbents were roughly the same, only PMMA 1 sorbent has a slightly lower sorption capacity (Table 2).

### 2.4. Zinc(II) Ions Sorption Studies—Isotherms

**Figure 6:**
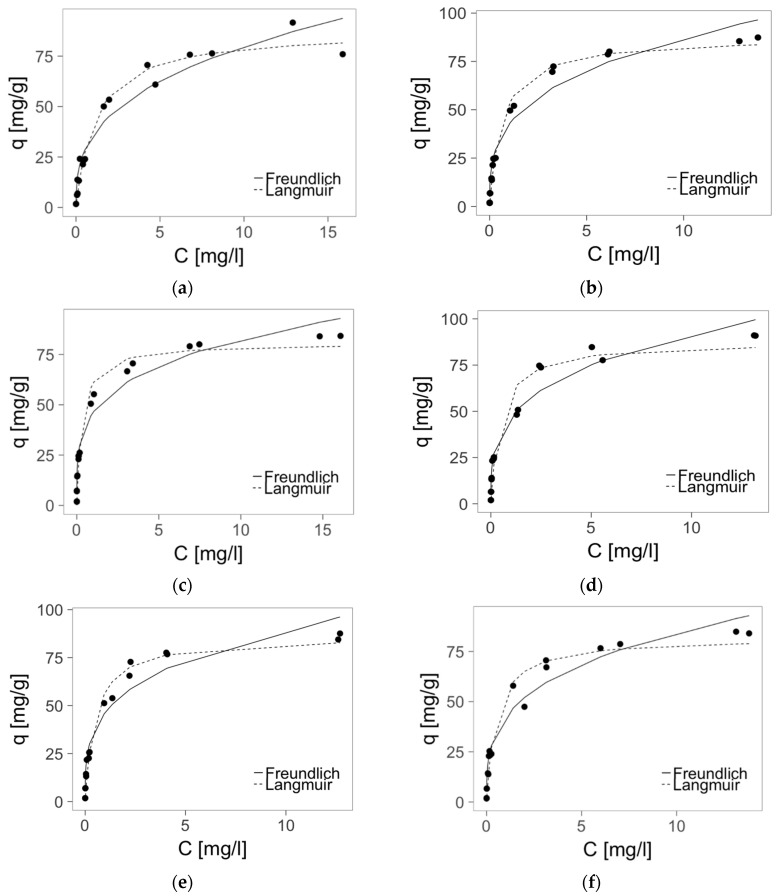
Adsorption isotherms of zinc on PMMA-based sorbents: (**a**) PMMA1; (**b**) PMMA2; (**c**) PMMA3; (**d**) PMMA4; (**e**) PMMA5; (**f**) PMMA6. Mass of sorbent 0.01 g, volume of solution: 0.025 L, pH = 6, temperature 22 ± 1 °C, time of contact 24 h.

Two isotherm equations—Langmuir and Freundlich—were tested in order to explain the adsorption of Zn(II) on PMMA-based sorbent. The isotherms and their parameters are presented in Figure 6 and Table 3, respectively.

The Langmuir model fits the sorption equilibrium data better (higher correlation coefficient, *R*^2^). Therefore, the parameters of this model were used for comparison of the studied materials sorption properties. Generally, the maximum sorption capacities were high (up to 87,5 mg/g) and did not depend on the origin of starting material (PMMA of various Mw) nor the kind of crosslinking ion (Ca, Mg). Only sorbents prepared from the waste PMMA of the lowest Mw had a little lower sorption capacities (slightly above 80 mg/g). However, these materials, especially crosslinked with Ca, had the highest B parameter, thus the strongest affinity to Zn(II) ions. The B parameter was used also for calculation of dimensionless constant separation factor RL [21], which indicates the character of sorption: RL > 1, unfavorable; RL = 1, linear; 0 < RL < 1, favorable; and RL = 0, irreversible. Our results (RL: 0.02–0.61; 0.01–0.47; 0.007–0.30; 0.01–0.39; 0.01–0.40; 0.01–0.40 for PMMA1; 2; 3; 4; 5 and 6, respectively) indicate that adsorption of zinc(II) ions on studied materials was favorable. Additionally, at Zn initial concentration above 6 mg/L, values of RL parameter were very low (<0.1), which suggests irreversible adsorption. 

Presented results show that waste PMMA may be an interesting raw material for the preparation of sorbents for zinc(II) ions removal. Using the presented method, it is possible to treat the PMMA blends, which contain other polymers or ceramic additives. Obtained sorbents have higher or similar sorption capacities towards Zn(II) ions (ca. 80 mg/g), compared to commercially available materials, e.g., Purolite C-100 MH (64.1 mg/g) [16], Amberlite IR-120 (87.7 mg/g) [17], Dowex 50W (19.7 mg/g) [18], Amberlite IRC-748 (28.1 mg/g) [19].

## 3. Materials and Methods

### 3.1. Materials

Poly(methyl methacrylate)s of *M*_w_: 350,000 and 996,000 g/mol were purchased from Sigma Aldrich (St. Louis, MO, USA). The waste poly(methyl methacrylate) (*M*_w_: 117,000 g/mol) was supplied from the landfill sites by Remondis (Tarnowskie Góry, Poland). The other reagents and standards such as: Diethylene glycol diethyl ether (Acros Organics), diethylene glycol methyl ether (Acros Organics), dimethyl sulfoxide (Acros Organics), deuterated dimethyl sulfoxide (Acros Organics), potassium hydroxide (Avantor, Poland), acetone (Chempur, Piekary Śląskie, Poland), potassium hydroxide (Avantor, Poland), sodium hydroxide (Avantor, Poland), hydrochloric acid ca. 35–37% (Chempur, Piekary Śląskie, Poland), nitric acid 65% (Suprapur, Merck, Germany), zinc nitrate (Avantor, Poland) and methanol (Acros Organics) were purchased and used without further purification. Zinc standard solution of 1 mg/mL was supplied by Merck. Deionized water was prepared using a Millipore Elix 10 system.

### 3.2. The Microcrystalline Suspension of Potassium Hydroxide (MSPH)

*MSPH* was obtained in a similar way to the method first described by *Morey and Smith* [22]. Potassium hydroxide flakes (20 g) were mixed with 50 mL of diethylene glycol diethyl ether (DEGDE) in a 100 mL conical flask adapted with an air condenser and argon gas inlet. Reaction vessel was stirred mechanically by collapsible teflon-coated stirring paddle at 300 rpm and heated up by silicon oil bath. Mechanical stirring was accelerated up to 1600 rpm as the solution temperature reached 140 °C. Moderate cooling rate (1.7 °C/min), along with vigorous stirring, were essential for obtaining microcrystalline suspension. Argon gas was purged through the air condenser, providing protection from the oxidation and the reaction with carbon dioxide. Suspension was cooled down to room temperature, and soon after that transferred to 50 mL conical flask with a silicon septum. Before each use, sample of MSPH was probed from stirred mixture, and the potassium hydroxide content was determined by colorimetric titration method.

### 3.3. Characterization of Waste Poly(methyl methacrylate)

The kind of polymeric waste was preliminary confirmed by the ATR-IR method. Then, the waste PMMA sample was dissolved in chloroform and the ceramic additives were filtered and analyzed by SEM (Phenom-World B.V., Eindhoven, The Netherlands) equipped with EDS detector. In the next step, the solvent was evaporated from the PMMA by rotatory evaporator (474 mbar, 40 °C). The non-ceramic additives were determined in the dried PMMA by ^1^H and ^13^C NMR. Finally, molecular weight and dispersity indices were determined by size-exclusion chromatograph (SEC) (Agilent Technologies, Santa Clara, CA, USA). For preparation of sorbent, waste PMMA was used as it was supplied, without further purification.

### 3.4. Analytical Methods

IR spectra were measured on a FTIR spectrophotometer (ATR method, Nicolet 6700, Thermo Fisher Scientific, Waltham, MA, USA). NMR spectroscopy: ^1^H and ^13^C NMR spectra were recorded in CDCl_3_ at concentrations of 20 mg/mL, operating at 400 and 75 MHz, respectively. Molecular weights and dispersity indices (Đ) were determined by size-exclusion chromatograph (SEC) equipped with an 1100 Agilent 1260 Infinity isocratic pump, autosampler, degasser, thermostatic box for columns and differential refractometer MDS RI Detector. Addon Rev. B.01.02 data analysis software (Agilent Technologies, Santa Clara, CA, USA) was used for data collecting and processing. The SEC calculated molecular weight was based on calibration applying linear polystyrene standards between 580 to 3,000,000 g/mol). Pre-column guard 5 μm (50 mm × 7.5 mm) and PLGel 5 μm MIXED-C (300 mm × 7.5 mm) columns were used for separation. The measurements were carried out in THF (HPLC grade) as the solvent at 40 °C with flow rate of 0.8 mL/min.

SEM micrographs of swollen and lyophilized (Christ Alpha 1-2 LDplus) sorbent were obtained utilizing scanning electron microscopy (Phenom Pro Desktop SEM). 

The concentration of zinc(II) during sorption studies was determined using ICP-AES spectrometer (ICP atomic emission spectrometer Varian 710-ES, Palo Alto, CA, USA).

### 3.5. Colorimetric Titration of Obtained Polymeric Matrix Samples (Hydrogel Acid Form)

Polymeric matrix sample (PM1, PM2 or PM3, 0.5 g, dry, acid form) was dispersed in 20 mL of 0.1 M NaOH, then 50 mL of demineralized water was added. In order to complete the reaction and swelling process, the overnight agitation by orbital shaker was applied. Then, phenoloftalein indicator (a few drops in alcoholic solution) was added and the excess of NaOH was titrated off with 0.1 M hydrochloric acid until total discoloration of solution.

### 3.6. General Procedure of Polymer Matrix Preparation from PMMA by the One-Pot Hydrolysis—Crosslinking Reaction

The poly(methyl methacrylate) (5 g) was dissolved in 40 mL of diethylene glycol diethyl ether (DEGDE) at a temperature of 105 °C. Obtained transparent and dense polymer solution was thoroughly purged with argon and cooled down to ambient temperature. Then, 5.9 g of the MSPH (equivalent of 25 mmol of potassium hydroxide) was added into polymer solution. The mixture was constantly stirred (250 rpm) using magnetic stirrer and the diethanoloamine (2.4 mL; 25 mmol), dimethyl sulfoxide (7.6 mL), as well as diethylene glycol methyl ether (0.5 mL; 4.3 mmol), were instilled into the polymer solution. Reaction vessels were heated up to 130 °C by oil bath, vapour pressure inside the vessels was diminished by thin, stainless needle in septum. After 35 min, reaction was quenched by oil bath removal and addition of demineralized water (70 mL). Then, liquid phase was discarded and product was obtained as a disk-like shape gel. The product was named “polymer matrix (PM)”. PM1, PM2, PM3 were obtained from poly(methyl methacrylate)s of *M*_w_: 996,000, 350,000 and 117,000 (waste) g/mol, respectively. 

### 3.7. General Procedure of Sorbents Preparation

Obtained product was immersed in a demineralized water for 12 h. After this time, liquid excess was sucked off on polyester mesh-cloth, and the completely swollen hydrogel was put into a beaker and fragmented by mechanical stirring at 1000 rpm. In the next step, the potassium salt of hydrogel was turned into the acid form. For this purpose, 0.5 M hydrochloric acid (100 mL, 0.05 mol) was added in excess. After 1 h, solid product was filtered and washed with 0.1 M hydrochloric acid (ca. 100 mL). This procedure was repeated using demineralized water until complete removal of chloride ions (silver ion test). The acid form of hydrogel was dried at 55 °C. Dry product was stirred overnight with 250 mL methanol-water mixture (25:1, V/V) to remove the non-crosslinked fractions and was filtered. Additionally, the hydrophobic and non-crosslinked fractions were extracted for 4 h with acetone (four times, 150 mL) using the Soxhlet’s apparatus. Pure acid form of hydrogel was dried to constant mass at 105 °C. The mass of dry, pure, crosslinked poly(methacrylic acid)-*co*-poly(methyl methacrylate) and hydrolysis degree was used for product yield calculation.

#### 3.7.1. Crosslinked Materials based on Poly(calcium methacrylate)-*co*-poly(methyl methacrylate)

Crosslinked samples of poly(methacrylic acid)-*co*-poly(methyl methacrylate) (2.0 g) were dispersed in 10 mL of demineralized water. Then, 100 mL of 2 M CaCl_2_ and 20 mL of 25% ammonia were added to the suspension, followed by stirring for 120 min at room temperature. Products were filtered, 20 mL of 2 M CaCl_2_ and 100 mL of demineralized water were added in order for complete saturation of the polymer’s matrices with calcium ions. Then, the crude sorbents were filtered off, stirred with demineralized water overnight, and washed to remove the chloride ions (silver nitrate test). Finally, the products were rinsed with 50 mL of ethanol, and dried to a constant mass at 105 °C. Dry and pure products were pulverised and sieved. The products were collected as the fractions between 0.125 mm and 0.250 mm. The products were named “sorbent” PMMA1, PMMA2, PMMA3 (obtained from polymer matrix PM1, PM2, PM3, respectively).

#### 3.7.2. Cross-Linked Poly(magnesium methacrylate)-*co*-poly(methyl methacrylate)

The crosslinked samples of poly(methacrylic acid)-*co*-poly(methyl methacrylate) (2.0 g) were dispersed in 30 mL of demineralized water, and 90 mL of 2 M NaOH was added. Suspension of swollen polymer beads was stirred for 120 min. Then, samples were filtered and washed with methanol until neutral pH (colorless phenolphthalein indication). Next, the hydrogel was being saturated with 100 mL of 1 M MgCl_2_ aqueous solution for 60 min. The crude sorbents, crosslinked poly(magnesium methacrylate)-co-poly(methyl methacrylate), were filtered, washed with water and methanol. Finally, sorbents were dried at 105 °C to constant mass. Dry products were pulverised and sieved. Fractions of 0.125–0.250 mm were used in further sorption studies. The products were named “sorbent” PMMA3, PMMA4, PMMA5 (obtained from polymer matrix PM1, PM2, PM3, respectively).

**Figure 7:**
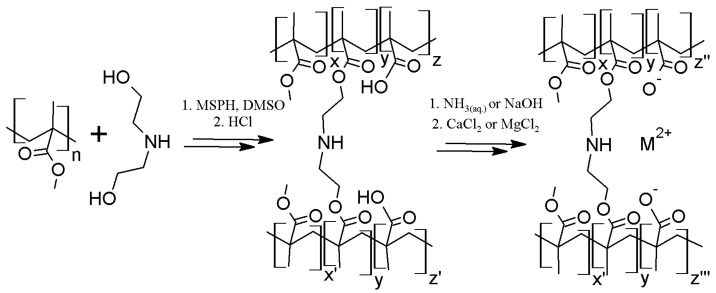
A scheme of reactions of the polymer matrices and sorbents preparation with their hypothetical structure. M^2+^—calcium or magnesium ion.

Overall procedure of preparation polymer matrices and sorbents described in Section 3.6 and Section 3.7 are shown in Figure 7.

### 3.8. Zinc(II) ions Sorption Studies—pH Dependence

Accurately weighed 0.01 g of studied sorbent was placed in 50 mL of zinc(II) nitrate solution (Zn: 30 mg/L) of pH adjusted with nitric acid or sodium hydroxide (1–7), then shaken using Incu-Shaker at room temperature (22 ± 1 °C) for 24 h. Next, the sorbent was separated from the solution using syringe filter (0.45 µm) and Zn(II) concentration in each solution was determined using ICP-AES method (ICP atomic emission spectrometer Varian 710-ES). Additionally, Ca(II) and Mg(II) concertation was also measured.

In all sorption studies, the sorption capacity of zinc(II) ions on studied PMMA-based sorbents (mg/g) was calculated using the formula:

*q* = (*c*_0_ − *c*) × *V*/*m*, (1)

where,

*c*_0_—the initial concentration of Zn(II) ions in the solution (mg/L),
*c*—the final concentration of Zn(II) ions in the solution (mg/L),
*V*—the volume of the solution (L),
*m*—the mass of the sorbent (g).

### 3.9. Zinc(II) ions Sorption Studies—Kinetics

The experiment was performed as described in pH-dependence Section 3.8, but 20 mL solution of pH = 6 was used and samples were filtered after 1, 3, 10, 15, 20, 30, 60, 120, 180, 240, 300, 360 and 480 min. 

Two elementary kinetic models (pseudo-first order and pseudo-second order) were utilized to analyze the adsorption kinetics of Zn(II):

the pseudo-first order equation [23,24]:

ln (*q*_m_ − *q*_t_) = ln *q*_m_ − *k*_1_ × *t*, (2)

the pseudo-second order equation [24]:

*t*/*q*_t_ = 1/(*k*_2_ × *q*^2^*m*)+ *t*/*q*_m_, (3)

where:

*q*_m_—the zinc(II) ions adsorbed on one gram of sorbent at equilibrium (adsorption capacity) (mg/g),
*q*_t_ —the zinc(II) ions adsorbed on one gram of sorbent at time “*t*” (mg/g),
*k*_1_—the rate constant of pseudo-first order adsorption model (1/min),
*k*_2_—the rate constant of pseudo-second order adsorption model (g/mg·min),
*t*—the time (min).

The model parameters were estimated by the least squares method to best fit the data.

### 3.10. Zinc(II) ions Sorption Studies—Isotherms

The experiment was performed as described in the pH-dependence Section 3.8, but 25 mL of solutions of pH = 6 of various concentration (1–50 mg/L) were used. 

Two elementary isotherms were utilized to analyze the equilibrium adsorption of Zn(II) ions: Langmuir isotherm [25]:

*q* = (*q*_m_ · *B* · *c*)/(1 + *B* · *c*), (4)

Freundlich isotherm [25]:

*q* = *K* · *c*^1/*n*^, (5)

where:

*q*—the zinc(II) ions adsorbed on one gram of sorbent at equilibrium (adsorption capacity) (mg/g),
*q*_m_—the maximum adsorption capacity (mg/g),
*B*—the equilibrium constant that corresponds to the adsorption energy (L/mg),
*c*—the equilibrium concentration of zinc(II) ions in the solution (mg/L),
*K*—[(mg/g)(L/mg)^1/n^] corresponds to the relative adsorption capacity,
*n*—corresponds to the adsorption intensity of the sorbent.

The model parameters were estimated by the least squares method to best fit the data.

Each result is an average value from three independent experiments.

## Figures and Tables

**Table 1:** PMMA hydrolysis-crosslinking reaction data.

Exp. no.	Polymer Matrix	PMMA Mw (by GPC) [*Da*]	Yield [%]	Degree of Hydrolysis [*%*]	Sorbent Sample no. Counter ion	
					Ca^2+^	Mg^2+^
1	PM 1	966,000	95	34	PMMA1	PMMA4
2	PM 2	350,000	96	32	PMMA2	PMMA5
3	PM 3	117,000	87	35	PMMA3	PMMA6

**Table 2:** Calculated parameters of pseudo-second and pseudo-first order adsorption kinetics model of zinc(II) ions on PMMA-based sorbents.

Sorbent	PMMA1	PMMA2	PMMA3	PMMA4	PMMA5	PMMA6
**Pseudo-first-order kinetics**						
*R*^2^	0.991	0.975	0.978	0.977	0.979	0.988
*q*_m_ [mg/g]	51.8 ± 0.9	52.2 ± 1.3	51.2 ± 1.1	54.6 ± 1.4	54.3 ± 1.4	53.6 ± 1.0
k_1_·10^2^ [1/min]	9.64 ± 0.68	15.51 ± 1.97	17.11 ± 1.96	9.40 ± 0.96	9.18 ± 0.90	12.30 ± 1.07
**Pseudo-second-order kinetics**						
*R*^2^	0.996	0.998	0.998	0.991	0.988	0.996
*q*_m_ [mg/g]	35.9 ± 2.2	42.5 ± 1.9	46.2 ± 1.6	46.8 ± 2.1	46.2 ± 2.1	42.3 ± 1.9
k_2_·10^4^ [g/mg∙min]	26.6 ± 2.0	44.3 ± 1.8	48.9 ± 2.2	23.1 ± 2.6	23.4 ± 2.9	33.5 ± 2.3

**Table 3:** Calculated parameters of isotherms, Langmuir and Freundlich, of Zn(II) ions sorption on PMMA-based sorbents; *q*_m_—the maximum adsorption capacity [mg/g], *B*—the equilibrium constant that corresponds to the adsorption energy [L/mg], *K* [(mg/g)(L/mg)^1/n^]—corresponds to the relative adsorption capacity, *n*—corresponds to the adsorption intensity of the sorbent.

Sorbent	PMMA1	PMMA2	PMMA3	PMMA4	PMMA5	PMMA6
**Calculated Parameters of Langmuir Isotherm**						
***R*^2^**	0.975	0.991	0.980	0.962	0.983	0.969
***q*_m_** **[mg/g]**	87.6 ± 3.5	87.6 ± 2.0	80.59 ± 2.6	87.50 ± 4.3	86.1 ± 3.0	81.8 ± 3.2
***B*** **[L/mg]**	0.84 ± 0.13	1.51 ± 0.15	3.06 ± 0.51	2.10 ± 0.52	1.96 ± 0.30	1.94 ± 0.37
**Calculated Parameters of Freundlich Isotherm**						
***R*^2^**	0.947	0.963	0.971	0.963	0.953	0.964
***K*** **[(mg/g)(L/mg)^1/n^]**	35.6 ± 2.3	42.5 ± 2.0	46.3 ± 1.7	47.1 ± 2.1	46.5 ± 2.1	42.3 ± 1.9
***n***	2.86 ± 0.25	3.21 ± 0.23	3.99 ± 0.26	3.45 ± 0.26	3.50 ± 0.29	3.34 ± 0.25
